# Deep learning-based prediction model of acute kidney injury following coronary artery bypass grafting in coronary heart disease patients: a multicenter clinical study from China

**DOI:** 10.3389/fcvm.2025.1600012

**Published:** 2025-06-23

**Authors:** Biao Hou, Tingting Liu, Pengyun Yan, Yuqing Wang, Xuejian Hou, Liang Li, Haiping Yang, Lin Chen, Taoshuai Liu, Kui Zhang, Shijun Xu, Yang Li, Ran Dong

**Affiliations:** ^1^ Department of Coronary Heart Disease Surgery, Beijing Anzhen Hospital, Capital Medical University, Beijing, China; ^2^ Capital Medical University, Beijing, China; ^3^ Department of Cardiac Surgery, The First Affiliated Hospital of Xi'an Jiaotong University, Xi'an, China; ^4^ Department of Cardiac Surgery, Jinan Third Hospital, Jinan, China; ^5^ Department of Cardiac Surgery, Handan First Hospital, Handan, China; ^6^ Department of Cardiac Surgery, Beijing Luhe Hospital, Capital Medical University, Beijing, China

**Keywords:** OPCABG, AKI, machine learning, XGBoost, CAD

## Abstract

**Introduction:**

Off-pump coronary artery bypass grafting (OPCABG) is an alternative to traditional coronary artery bypass grafting (CABG), which avoids cardiopulmonary bypass. However, acute kidney injury (AKI) is a common complication, with incidence rates ranging from 5% to 42%, significantly affecting postoperative outcomes. This study aimed to develop a robust risk prediction model for post-OPCABG AKI using machine learning (ML) techniques.

**Methods:**

We conducted a multicenter, retrospective study involving 3,043 coronary artery disease (CAD) patients, with an overall AKI incidence of 15.28%. The cohort was divided into a training set (*n* = 2,130) and a validation set (*n* = 913). An external validation cohort of 878 patients was also included. Five ML methods -Support Vector Machine (SVM), Decision Tree (DT), Random Forest (RF), AdaBoost, and XGBoost-were employed to predict the risk of AKI.

**Results:**

The XGBoost model demonstrated the highest performance, with an area under the curve (AUC) of 0.88, sensitivity of 82%, and specificity of 83% in the internal validation set. In the external validation cohort, the XGBoost model achieved an AUC of 0.84, sensitivity of 74%, and specificity of 90%. The model utilized 26 predictive features, including patient demographics and preoperative laboratory values.

**Discussion:**

The XGBoost model outperformed other ML methods (SVM, DT, RF, and AdaBoost) in both internal and external validations, demonstrating its robustness and generalizability. By integrating diverse patient data from multiple institutions, our model significantly improved AKI risk assessment and identified novel predictive factors. These findings highlight the potential of machine learning models in enhancing AKI risk prediction and supporting personalized management strategies to improve outcomes in OPCABG patients.

## Introduction

Coronary artery bypass grafting (CABG) is a widely performed surgical procedure aimed at improving myocardial blood flow and relieving symptoms of angina in patients with coronary artery disease (CAD) (1) However, despite advancements in surgical techniques and postoperative care, acute kidney injury (AKI) remains a significant and common complication following CABG, with prevalence rates ranging from 5% to 42% (2, 3). AKI adversely impacts postoperative outcomes, leading to increased morbidity and mortality, longer ICU and hospital stays, and higher healthcare costs (4). As such, identifying patients at high risk of AKI is essential for improving prognosis and outcomes in clinical practice (5).

In recent years, off-pump coronary artery bypass grafting (OPCABG), a procedure that avoids the use of cardiopulmonary bypass, has been introduced as an alternative to traditional CABG. While OPCABG has been associated with reduced inflammatory responses and better early recovery, it also carries its own set of risks, including AKI. The incidence of AKI following OPCABG remains a concern, as it can significantly affect patient outcomes, including longer hospitalization, increased healthcare costs, and higher mortality rates (6, 7). Therefore, accurately predicting AKI risk in OPCABG patients is of paramount importance for improving postoperative care and enhancing recovery.

Several risk prediction models for AKI following cardiac surgery, such as the KDIGO guidelines and the Cleveland score, have been developed. These models assess AKI risk based on serum creatinine changes, urine output, and preoperative variables. However, these models are primarily derived from broad cardiac surgery cohorts and are based on single-center studies, limiting their applicability to specific populations such as those undergoing OPCABG. As a result, their predictive accuracy tends to be lower when applied to isolated CABG or OPCABG cohorts. For instance, the KDIGO guidelines have an area under the curve (AUC) of 0.74 in mixed cardiac surgery populations but only 0.67 in CABG-specific cohorts (8). Similarly, the Cleveland score has an AUC of 0.75 in broad cardiac surgery populations but only 0.68 in CABG patients (9). Furthermore, existing models often fail to consider surgery-specific factors that play a crucial role in AKI risk for OPCABG patients, such as graft type, ischemic time, and cardiopulmonary bypass time. Incorporating these factors has been shown to improve predictive accuracy significantly (10).

In light of these limitations, machine learning (ML) and deep learning (DL) techniques present a promising avenue for improving the prediction of AKI in OPCABG patients. ML algorithms, such as Support Vector Machine (SVM), Decision Tree (DT), Random Forest (RF), AdaBoost, and XGBoost, can analyze large, multidimensional datasets and identify complex patterns that are difficult to detect using traditional statistical methods (11). Among these, XGBoost has gained particular attention due to its robust performance in predictive modeling tasks, its ability to handle missing data efficiently, and its regularization techniques that prevent overfitting (12).

This study aims to develop and validate a deep learning-based prediction model specifically designed to assess the risk of AKI following OPCABG in patients with coronary heart disease (CHD). By utilizing multicenter data from clinical settings across China, we seek to create a robust and scalable model that can provide individualized, precise risk assessments for AKI in OPCABG patients. Our goal is to integrate this model into clinical practice, facilitating better decision-making and ultimately improving patient outcomes. This approach will also offer insights into the underlying mechanisms of AKI in the OPCABG population, contributing to the development of more targeted and personalized management strategies for at-risk patients.

In conclusion, this study endeavors to fill the gap in current AKI prediction models by developing a specialized, data-driven model tailored to the OPCABG population. By leveraging the power of machine learning, we aim to enhance the accuracy, generalizability, and clinical applicability of AKI risk prediction for patients undergoing OPCABG surgery, thereby improving postoperative care and patient outcomes.

## Materials and methods

### Study population and selection criteria

Between January 2022 and January 2024, 4,283 patients who underwent OPCABG at our hospital were screened. Patients were excluded if: (1) age <18 years or >80 years; (2) Patients with preoperative renal dysfunction (Scr >133 µmol/L); (3) underwent repeat OPCABG surgery; (4) had concurrent other surgeries; (5) had a history of cardiac surgery; (6) had no available medical records; and to ensure the completeness of clinical data for all enrolled patients, those with missing or incomplete clinical information were excluded. This approach was taken to minimize potential bias and the impact of missing data on the results. Ultimately, 3,491 patients were included and randomly assigned to the model training and internal validation groups in a 7:3 ratio.

**Figure 1:**
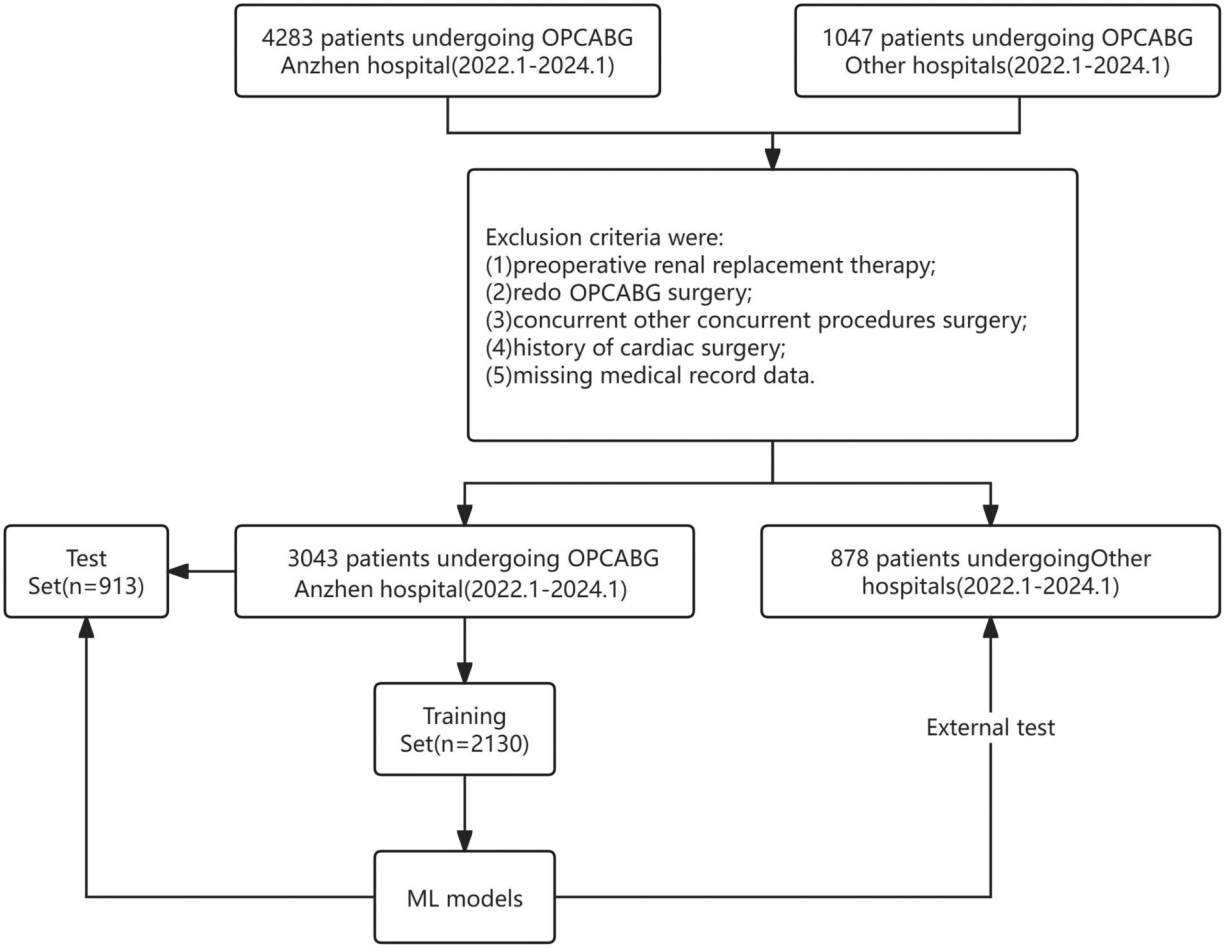
Flowchart of patient selection.

Additionally, 1,047 patients from four other medical centers were screened. Following the same exclusion criteria, 878 patients were enrolled and assigned to the external validation group to validate the performance of the ML models. The patient selection process is illustrated in Figure 1.

This multicenter retrospective study was approved by the Ethics Review Committee of hospital. The requirement for written informed consent was waived due to the retrospective design of the study.

### Definition and outcome

AKI was defined according to the Kidney Disease Improving Global Outcomes (KDIGO) criteria, which consider serum creatinine levels and urine output alterations. Specifically, AKI was identified by an increase in serum creatinine level ≥0.3 mg/dl within 48 h, a rise to ≥1.5 × baseline within seven days, or a urine output <0.5 ml/kg/hour for six hours. Owing to the administration of diuretics and challenges in collecting clinical records, urine output was not utilized for AKI diagnosis preoperatively or postoperatively. The primary outcome of this study was AKI development after OPCABG.

### Variable selection

The variable selection process in this study was based on the European System for Cardiac Operative Risk Evaluation II (EuroSCORE II) as a reference (13). EuroSCORE II is a widely used predictive model for assessing cardiac surgical risk, evaluating clinical characteristics and surgical risk factors, such as age, sex, cardiac functional status, history of chronic diseases, acute conditions, and type of cardiac surgery. This model has been extensively validated and is effective in predicting mortality risk following OPCABG, including in Chinese patients. In this study, 26 variables were selected and extracted from hospital electronic medical records, primarily derived from the EuroSCORE II model. However, additional variables, including calcium and HDL-C, were incorporated due to their potential relevance in predicting AKI risk following OPCABG. These variables, along with those from EuroSCORE II, were refined through feature selection techniques to ensure optimal model performance.

### Statistical analysis

Continuous variables were expressed as mean ± standard deviation (SD) or median with interquartile range (IQR). The Students *t*-test or Mann–Whitney *U* test was used to compare the differences between groups according to the data distribution. Categorical variables were reported as numbers and percentages (%), and the differences between groups were compared using Pearsons chi-square test or Fishers exact test, as appropriate.

Using the default hyperparameters, predictive models were initially constructed using several ML techniques, including SVM, DT, RF, AdaBoost, and XGBoost. These models were created as baseline models for the task of predicting AKI after OPCABG. After constructing these initial models, we optimized the hyperparameters to enhance model performance and reliability through a grid search approach. The grid search allowed for the systematic exploration of multiple hyperparameter combinations, ensuring that the model configurations were carefully adjusted to achieve the best possible predictive accuracy.

To prevent overfitting and ensure robust model generalization, we applied ten-fold cross-validation. This technique involved partitioning the training dataset into ten subsets, or folds. For each iteration, nine of the folds were used to train the model, while the remaining fold served as the validation set. This process was repeated ten times, with each fold being used as the validation set once. Cross-validation helped us ensure that the models were not overly dependent on specific subsets of the data, thus providing a more accurate estimate of their generalization capabilities.

The models were evaluated by comparing several key performance metrics, including the Area Under the Curve (AUC), Brier score, Net Reclassification Improvement (NRI), Integrated Discrimination Improvement (IDI), Sensitivity, Specificity, Positive Predictive Value (PPV), and Negative Predictive Value (NPV). These metrics allowed us to assess the models' ability to discriminate between patients at high and low risk of AKI, as well as their overall predictive accuracy. By analyzing these metrics, we selected the best-performing model, ensuring reliable and clinically relevant predictions for AKI risk after OPCABG surgery.

Additionally, SHAP values were used to identify critical factors influencing OPCABG-AKI risk, highlighting the importance of individual features in the model output. However, the use of NRI and IDI requires clearer explanation. NRI measures how well a new model reclassifies subjects compared to an old model, specifically evaluating improvements in prediction accuracy for individuals who experience an event vs. those who do not. IDI quantifies the improvement in risk prediction by comparing the difference in average predicted probabilities between those with and without the event for old and new models. We also evaluate internal and external validation through calibration curves. By employing NRI and IDI, we can more comprehensively assess the performance enhancements provided by our model beyond traditional metrics like AUC. SHAP values provide a detailed measure of feature importance by quantifying the contribution of each feature to the model's predictions, considering all possible combinations of feature values. This approach helped us identify and highlight critical factors influencing OPCABG-AKI risk, thereby improving the transparency and interpretability of the model's output.

All statistical analyses were performed using Python (version 3.11) and SPSS (version 27). Specifically, in Python, we used several libraries to support our machine learning and explainability efforts, including xgboost for implementing the XGBoost algorithm, and the shap library for calculating SHAP values to interpret the model outputs. Statistical significance was defined as a two-tailed *P*-value of < 0.05.

## Results

### Baseline characteristics

The training set consisted of 2,130 cases with an average age of 62.4 ± 8.78 years, including 1,587 males (74.54%), and 326 cases (15.31%) developed AKI. The internal validation set included 913 cases with an average age of 63.19 ± 8.53 years, consisting of 667 males (73.06%), and 139 cases (15.22%) developed AKI. Due to random assignment, there were no significant differences in major clinical characteristics between the two groups (Table 1). The external validation set included 878 cases with an average age of 63.75 ± 8.29 years, including 632 males (71.98%), and 136 cases (15.49%) developed AKI. Patient baseline characteristics are shown in Supplementary Table S1. The 26 selected features are shown in Table 1.

**Table 1:** Characteristics of modeling group and validation groups.

Variables	Training dataset	Internal validation	*P* value	External validation dataset	*P* value
Number	2,130	913		878	
Age (years)	62.4 ± 8.78	63.19 ± 8.53	0.612	63.75 ± 8.29	0.73
Male (*n*, %)	1,587 (74.54)	667 (73.06)	0.391	632 (71.98)	0.275
Hypertension (*n*, %)	1,421 (66.74)	618 (67.69)	0.614	555 (63.22)	0.143
Diabetes (*n*, %)	825 (38.75)	376 (41.18)	0.21	398 (45.33)	0.007
CBP (*n*, %)	377 (17.71)	167 (18.29)	0.756	128 (14.58)	0.074
Angina (*n*, %)	1,942 (91.22)	838 (91.79)	0.725	808 (92.03)	0.799
BMI (kg/m^2^)	25.78 ± 3.2	25.68 ± 3.16	0.252	26.08 ± 3.28	0.221
Preoperative Scr	74.68 ± 29.03	75.18 ± 28.97	0.421	71.24 ± 30.14	0.245
Preoperative LVEF	58.43 ± 9.17	58.06 (9.53)	0.158	58.63 ± 8.69	0.108
TG	1.63 ± 1.06	1.68 ± 0.93	0.37	1.66 ± 1.27	0.143
TC	3.93 ± 1.01	3.98 ± 1.05	0.8	3.97 ± 1.03	0.617
Hemoglobin	116.96 ± 23.38	117.39 ± 23.53	0.522	111.93 ± 24.2	0.325
HDL-C	0.97 ± 0.23	1.0 ± 0.24	0.248	1.06 ± 0.56	0.001
LDL-C	2.31 ± 0.84	2.33 ± 0.89	0.265	2.43 ± 0.98	0.009
Urea	6.82 ± 3.32	6.96 ± 3.31	0.296	6.55 ± 2.76	0.105
UA	335.13 ± 89.94	333.07 ± 96.67	0.219	332.74 ± 97.75	0.009
TP	68.43 ± 5.62	68.48 ± 5.64	0.593	67.45 ± 6.23	0.001
T-Bil	12.33 ± 5.9	12.03 ± 5.45	0.223	12.61 ± 6.61	0.201
D-Bil	3.92 ± 2.33	3.86 ± 2.04	0.175	4.23 ± 3.02	0.025
WBC	9.95 ± 4.08	9.75 ± 3.92	0.148	9.92 ± 4.62	0.876
RBC	3.81 ± 0.75	3.84 ± 0.77	0.403	3.68 ± 0.81	0.029
PLT	211.15 ± 75.03	214.16 ± 78.44	0.269	210.42 ± 76.4	0.448
Neu	74.26 ± 12.64	73.79 ± 12.73	0.296	74.81 ± 13.2	0.198
Glu	8.15 ± 3.46	8.17 ± 3.42	0.828	7.95 ± 3.47	0.235
Ca	2.23 ± 0.16	2.23 (0.16)	0.188	2.27 ± 1.03	0.071
ALP	82.24 ± 36.78	81.94 ± 30.26	0.703	81.16 ± 68.23	0.053
AKI	326 (15.31%)	139 (15.22%)	0.762	136 (15.49%)	0.812

CVD, cardiovascular disease; CBP, chronic bronchitis; COPD, chronic obstructive pulmonary disease; BMI, body mass index; Preoperative Scr, Preoperative serum creatinine; Preoperative LVEF, preoperative left ventricular ejection fraction; TG, Triglycerides; TC, total cholesterol; HDL-C, high-density lipoprotein cholesterol; LDL-C, low-density lipoprotein cholesterol; UA, uric acid; Alb, albumin; TP, total protein; T-Bil, total bilirubin; D-Bil, direct bilirubin; WBC, white blood cell count; RBC, red blood cell count; PLT, platelet count; Neu, neutrophil count; Glu, Glucose; Ca, Calcium; ALP, Alkaline Phosphatase; AKI, Acute Kidney Injury.

### Model performance

For the internal dataset, we evaluated five machine learning models: Support Vector Machine (SVM), Decision Tree (DT), Random Forest (RF), AdaBoost, and XGBoost. The performance of these models was assessed using area under the curve (AUC) metrics, as shown in Supplementary Figure S1, and the results are summarized in Table 2. The XGBoost model demonstrated the best overall performance with the highest AUC of 0.885, compared to 0.811 for SVM, 0.765 for DT, 0.849 for RF, and 0.849 for AdaBoost. The XGBoost model also exhibited superior sensitivity (80.2%) and specificity (89.2%), highlighting its robust predictive ability. In contrast, the Decision Tree (DT) model displayed the lowest AUC, with a sensitivity of 69.4% and specificity of 87.8%. Table 2 provides a comprehensive overview of the performance metrics for each model.

**Table 2:** Performance of each ML model in the training dataset.

Model	AUC	Sensitivity	Specificity	PPV	NPV	Accuracy	Recall	F1 Score	Brier score
SVM	0.81	0.59	0.83	0.72	0.88	0.88	0.53	0.61	0.22
DT	0.76	0.99	0.54	0.31	0.89	0.63	0.92	0.45	0.23
RF	0.85	0.69	0.87	0.9	0.91	0.72	0.78	0.61	0.20
AdaBoost	0.86	0.14	0.89	0.88	0.82	0.90	0.68	0.71	0.17
XGBoost	0.88	0.80	0.89	0.96	0.95	0.94	0.81	0.87	0.14

AUC, area under the curve; PPV, positive predictive value; NPV, negative predictive value.

In terms of discriminative power, the XGBoost model showed notable results, achieving an accuracy of 94.083%, an F1 score of 0.87, and a recall rate of 0.81. Additionally, the Brier scores for the models were 0.22 for SVM, 0.23 for DT, 0.20 for RF, 0.17 for AdaBoost, and 0.14 for XGBoost, confirming that XGBoost outperformed the other models. The Hosmer-Lemeshow goodness-of-fit test further supported this, indicating that the calibration of the XGBoost model was optimal, as shown in Supplementary Figure S2. These findings indicate that the XGBoost model has the best predictive ability and clinical utility among the models evaluated. Consequently, the XGBoost model was selected as the optimal model for further analysis.

### Validation using optimal covariates

**Figure 2:**
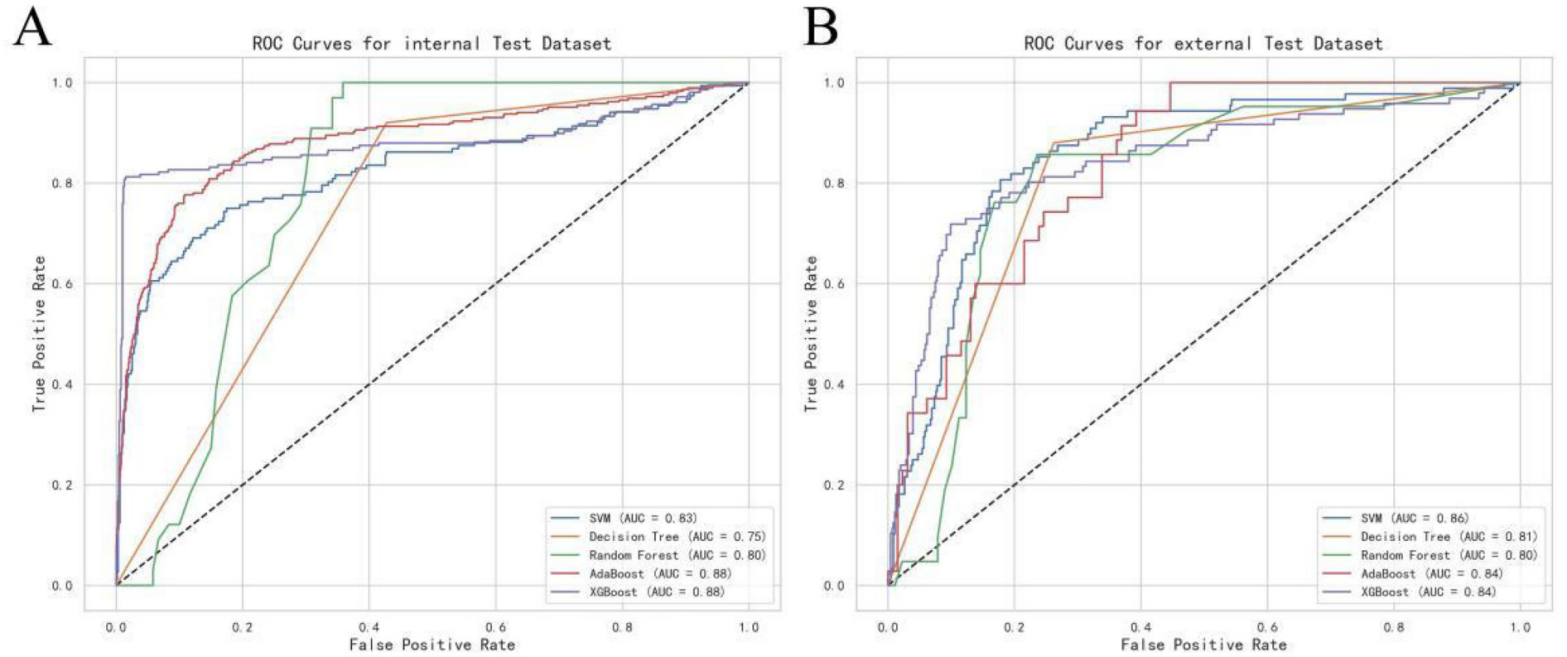
Receiver operating characteristic (ROC) curve of the risk assessment model: **(A)** Internal test group. **(B)** External test group.

Using the optimal covariates, we validated the XGBoost model in both the internal and external validation datasets. In the internal validation set, the AUC values for the different models were 0.83 (95% CI: 0.80–0.86) for XGBoost, 0.75 (95% CI: 0.71–0.79) for SVM, 0.80 (95% CI: 0.77–0.83) for RF, 0.88 (95% CI: 0.85–0.91) for AdaBoost, and 0.88 (95% CI: 0.85–0.91) for DT (Figure 2A). In the external validation set, the AUC values were 0.86 (95% CI: 0.82–0.91) for XGBoost, 0.81 (95% CI: 0.77–0.85) for SVM, 0.80 (95% CI: 0.77–0.83) for RF, 0.84 (95% CI: 0.81–0.87) for AdaBoost, and 0.84 (95% CI: 0.81–0.87) for DT (Figure 2B). Specifically, in the internal validation set, the XGBoost model achieved a specificity of 82.8% and sensitivity of 81.8%. In the external validation set, it achieved a specificity of 89.9% and sensitivity of 74.7% (Table 3). These results confirm that the XGBoost model consistently outperformed the other models in both internal and external validation sets.

**Table 3:** Performance of each ML model in the validation dataset.

Dataset	Model	AUC	Sensitivity	Specificity	PPV	NPV	95%CI (AUC)
Internal	SVM	0.83	0.75	0.83	0.48	0.94	[0.80, 0.86]
Internal	DT	0.75	0.86	0.57	0.31	0.95	[0.71, 0.79]
Internal	RF	0.80	0.86	0.69	0.46	0.94	[0.77, 0.83]
Internal	AdaBoost	0.88	0.72	0.89	0.61	0.93	[0.85, 0.91]
Internal	XGBoost	0.88	0.82	0.83	0.68	0.92	[0.85, 0.91]
External	SVM	0.86	0.81	0.82	0.46	0.96	[0.83, 0.89]
External	DT	0.81	0.86	0.69	0.29	0.97	[0.77, 0.85]
External	RF	0.80	0.85	0.76	0.46	0.95	[0.77, 0.83]
External	AdaBoost	0.84	0.84	0.63	0.44	0.91	[0.81, 0.87]
External	XGBoost	0.84	0.74	0.90	0.60	0.94	[0.81, 0.87]

AUC, area under the urve, PPV, positive predictive value, NPV: negative predictive value.

### Analysis of key contributors to the predictive model

**Figure 3:**
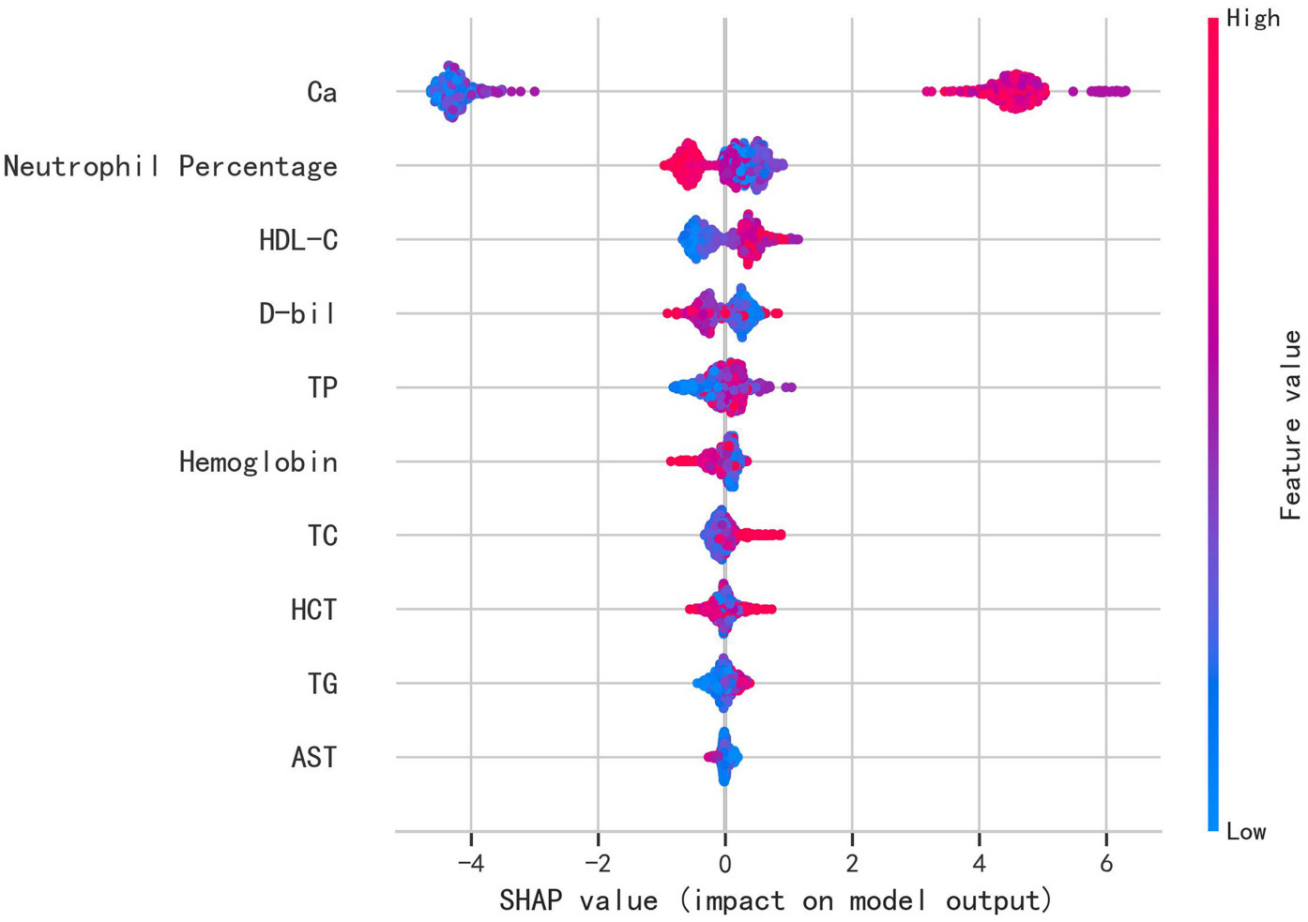
Shapley additive exPlanations (SHAP) values of variables.(Ca, total calcium; HDL-C, high-density lipoprotein cholesterol; D-bil, direct bilirubin; TP, total protein; hemoglobin; TC, total cholesterol; HCT, hematocrit; TG, triglycerides; AST, aspartate aminotransferase).

To identify the key contributors to the predictive model, we used SHAP (Shapley Additive Explanations) summary plots to highlight the top ten features in the XGBoost model. These features were ranked based on their importance, providing a clear visual representation of their impact on the model's predictions. Dependency plots were also generated to show how individual input features affected the final prediction outcomes of the XGBoost model (Figure 3). A SHAP value greater than zero indicates an increased risk of AKI, allowing for the identification of risk factors that contribute to a higher likelihood of AKI.

The top ten most influential features identified by the model were: Calcium (Ca), neutrophil percentage, HDL-C, Direct Bilirubin (D-bil), Total Protein (TP), hemoglobin, Total Cholesterol (TC), hematocrit (HCT), triglycerides (TG), and Aspartate Aminotransferase (AST). These features align with previous studies and established clinical knowledge, emphasizing their role in predicting AKI after CABG surgery (14, 15). Notably, some features that are typically overlooked in clinical practice, such as laboratory values and demographic factors, emerged as important predictors, offering new insights into AKI risk factors.

**Figure 4:**
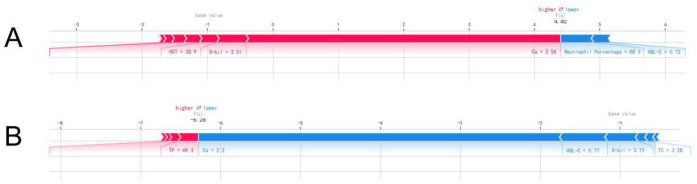
The individual SHAP force plots for patients who **(A)** suffered postoperative AKI and **(B)** did not suffer postoperative AKI.

The SHAP values were used to distinguish risk factors for patients who did or did not suffer from postoperative AKI. For example, higher values of certain features such as calcium, hemoglobin, and HDL-C were associated with a decreased risk of AKI, while higher values of factors such as triglycerides and neutrophil percentage indicated a higher risk of AKI (Figure 4). The color coding of SHAP values (red for factors increasing risk and blue for factors decreasing risk) allowed for a more intuitive understanding of how each variable impacted the final AKI prediction.

Overall, the identification of these key features, particularly those previously not emphasized in clinical practice, underlines the value of using advanced machine learning techniques to gain deeper insights into the complex factors influencing AKI risk. By focusing on both well-established and novel predictors, the XGBoost model enhances its predictive accuracy and clinical relevance, providing a more comprehensive understanding of the underlying mechanisms of AKI in post-OPCABG patients. This improvement in prediction accuracy can guide more targeted interventions and personalized care strategies for patients at high risk of AKI following OPCABG.

### Further validation of model performance

To further validate the performance of our model, we employed two statistical metrics: the Net Reclassification Improvement (NRI) and the Integrated Discrimination Improvement (IDI). These metrics were used to evaluate the enhancement in predictive performance when comparing the XGBoost model with other machine learning models, including SVM, DT, RF, and AdaBoost.

In the internal validation set, the NRI values for the XGBoost model were 0.5852 (95% CI: 0.3651–0.8053), 0.8988 (95% CI: 0.6860–1.1116), and 0.6847 (95% CI: 0.4665–0.9028), respectively, for SVM, DT, RF, and AdaBoost. Similarly, the IDI values for the XGBoost model were 0.0144 (95% CI: −0.0022–0.0311), 0.1632 (95% CI: 0.1168–0.2097), and 0.1082 (95% CI: 0.0708–0.1455). In the external validation set, the XGBoost model showed significant improvement over the other four machine learning models, as detailed in Table 4.

**Table 4:** Comparison of NRI and IDI between XGBoost model and the other four models.

Model	NRI-Internal	IDI-Internal	NRI-External	IDI-External
XGBoost-SVM	0.131578951	0.392569959	0.212936611	0.407685267
XGBoost-DT	0.504385916	0.495297804	0.323285899	0.41522068
XGBoost-RandomForest	0.144145712	0.200742565	0.211148416	0.20303275
XGBoost-AdaBoost	0.254385965	1.175069473	0.183699871	1.230715464

NRI, net reclassification improvement; IDI, integrated discrimination improvement.

These results demonstrate the enhanced predictive capability of the XGBoost model compared to the other models, indicating its robustness and reliability across both internal and external validation sets. The use of NRI and IDI further confirms the superior performance of the XGBoost model in predicting AKI following OPCABG surgery in patients with coronary heart disease, emphasizing its potential for improving risk prediction in clinical practice.

## Discussion

In this study, we present a deep learning-based prediction model for Acute Kidney Injury (AKI) following OPCABG in CHD patients, with a focus on a multicenter cohort from China. Our model focus on OPCABG patients and the inclusion of advanced feature selection techniques. In comparison to earlier models, such as those utilizing traditional machine learning methods (e.g., SVM and decision trees) (16), our deep learning approach provides a more robust handling of complex, high-dimensional data, improving predictive accuracy. Then we compared the effectiveness of five machine-learning methods (SVM, DT, RF, AdaBoost, and XGBoost) in predicting the risk of AKI following OPCABG. The results indicate that XGBoost outperformed the other models across various metrics.

When comparing our model's performance to prior studies, such as those by Hyung-Chul et al. (17) and Thongprayoon et al. (18), which used traditional machine learning methods and smaller datasets, our deep learning model outperforms in terms of both sensitivity and specificity for AKI prediction in OPCABG patients. Furthermore, our model addresses the unique characteristics of the Chinese patient population, whose demographic and clinical features may differ significantly from those in Western cohorts.

Serum creatinine has long been used as a marker for kidney function, but it has limitations, particularly in the early detection of AKI. Elevated serum creatinine levels are often a late indicator of kidney damage, and they may not reflect acute changes in kidney function immediately after surgery. This can result in delayed diagnosis and missed opportunities for early intervention. In contrast, the ML model developed in this study integrates a variety of patient data and clinical parameters, enabling it to predict AKI much earlier, often before serum creatinine levels become elevated. By capturing complex relationships between multiple factors, such as patient demographics, comorbidities, and preoperative variables, the ML model demonstrates a superior ability to identify high-risk patients for AKI. This early prediction is crucial for timely preventive measures, reducing the risk of serious complications.

XGBoost achieved the highest accuracy, significantly surpassing the other four methods and demonstrating a superior overall predictive capability. In terms of sensitivity, XGBoost was superior in correctly identifying patients who developed AKI, indicating its effectiveness in detecting high-risk patients. Regarding specificity, XGBoost performed exceptionally well in excluding patients who did not develop AKI, showing a lower false-positive rate. Notably, XGBoost had the highest AUC value, reflecting an optimal balance between the sensitivity and specificity.

XGBoost exhibited outstanding performance metrics, which can be attributed to several inherent characteristics of the algorithm. First, XGBoost utilizes a gradient boosting framework that allows the optimization of any differentiable loss function, making it highly effective in handling complex medical data with non-linear relationships. Second, XGBoost incorporates regularization techniques to prevent overfitting, which enhances the generalizability of the model across multicenter data (19). While SVM, Decision Tree, Random Forest, and AdaBoost each have their strengths, they underperformed compared to XGBoost in this study. Although powerful in binary classification tasks, SVM struggles in high-dimensional feature spaces and requires extensive hyperparameter tuning (20). Decision Tree models, despite their interpretability, Decision Tree models tend to overfit and lack the predictive power needed for complex datasets (21). Random Forest, as an ensemble method, offers greater stability but falls short in precision and recall compared to XGBoost. Similarly, AdaBoost, while improving over single Decision Trees, is sensitive to noisy data and outliers, leading to inferior performance compared to XGBoost (22).

The superior performance of XGBoost in predicting AKI risk after AKI post-OPCABG has significant clinical implications. Early and accurate identification of high-risk patients can facilitate timely medical intervention, thereby improving patient outcomes and reducing healthcare costs. The ability of XGBoost to handle large-scale and heterogeneous datasets makes it particularly valuable in multicenter settings, where it can accommodate the wide variability in patient populations and clinical practices. Integrating XGBoost-based predictive models into clinical decision support systems can enhance the precision of risk stratification and enable personalized patient care.

Timely and accurate prediction of AKI after OPCABG is crucial for identifying high-risk patients and promptly implementing preventive interventions. This can significantly reduce the incidence and mortality rates of post-OPCABG AKI. Recent studies have shown that ML methods have great potential for predicting AKI after OPCABG. For instance, Zhang et al. utilized an SVM algorithm to develop an ML model that effectively identified high-risk postoperative patients (23). Similarly, KHANH et al. employed recurrent neural networks (RNN) to create an ML model whose predictive performance surpassed that of traditional clinical scoring systems (24). Additionally, Hou et al. used the random forest (RF) algorithm to successfully construct a predictive model, which demonstrated high efficiency and reliability on a large-scale patient dataset (14).

However, most of these ML models were developed under conditions of limited data and computational resources, which constrained their ability to comprehensively explain their predictive mechanisms. In this study, we compared and contrasted several ML methods, including SVM, DT, RF, AdaBoost, and XGBoost to determine the model with optimal accuracy and discrimination in predicting post-OPCABG AKI. We found that the XGBoost model performed the best in terms of predictive performance. By utilizing SHAP values and LIME techniques, we were able to elucidate the main drivers of the model predictions, thereby enhancing the interpretability of the XGBoost model.

Interpretable ML models, particularly the XGBoost model, have significant clinical importance for predicting AKI following OPCABG. Early detection of AKI is vital for timely intervention and improvement in patient outcomes. Transparency and interpretability achieved through techniques such as LIME increase clinical trust and willingness to use these models. Identifying the key influencing factors, such as patient age, preoperative kidney function, provides critical insights into the risk factors for post-OPCABG AKI (25).

Moreover, integrating these ML models into clinical practice can facilitate personalized medicine, enabling clinicians to tailor interventions based on individual patient-risk profiles (26). Future research should focus on expanding the dataset and incorporating additional clinical variables to further enhance the model's robustness and generalizability (27). The ongoing collaboration between data scientists and clinicians is essential to refine these predictive models and ensure their seamless integration into clinical workflows.

These findings underscore the multifactorial nature of AKI risk in cardiac surgery patients and highlight the need for a comprehensive approach to its prediction and prevention. Integrating these factors into ML models can enhance their predictive accuracy and utility in the clinical setting (23). Collaboration between data scientists and clinicians is essential for refining these models and developing practical tools for real-time decision support.

While our study provides a comprehensive technical comparison of the XGBoost model and highlights its predictive capabilities using SHAP analysis, it is crucial to contextualize these findings in terms of their clinical implications. The identification of clinically relevant features such as age, baseline serum creatinine levels, and history of hypertension and diabetes aligns with established medical knowledge, affirming the model's reliability in a clinical context.

However, the emergence of unexpected predictors, such as specific laboratory values and demographic factors, necessitates further clinical investigation. Understanding these novel predictors could uncover new insights into AKI risk factors, potentially leading to improved patient management and preventative strategies.

Mechanistically, the features included in our model, such as calcium levels, neutrophil count, and chronic bronchitis, are closely linked to AKI pathophysiology. Elevated calcium levels, for instance, play a direct role in renal function and are associated with renal vasoconstriction and endothelial injury (28, 29). Neutrophils, as inflammatory markers, contribute to renal damage through their role in the inflammatory cascade (30). Chronic bronchitis, a respiratory condition, can exacerbate renal injury due to systemic inflammation and hypoxemia, which may impair renal perfusion.

To bridge the gap between technical findings and clinical application, future research should focus on validating these predictors in diverse clinical settings and translating model insights into practical guidelines for clinicians. This approach will ensure that the model's technical strengths are effectively harnessed to enhance patient care and outcomes.

### Limitations

Although the results are encouraging, this study has several limitations. The retrospective nature of the data may have introduced biases that could affect the model's performance, such as selection bias and information bias. Additionally, while XGBoost demonstrates strong predictive capabilities, the interpretability of the model remains a challenge. This lack of transparency can hinder clinicians' trust and acceptance of the model's predictions.

Another limitation is the potential for overfitting, despite the use of cross-validation techniques. The model may perform well on the training and validation datasets but might not generalize effectively to new, unseen data. This issue underscores the need for rigorous prospective validation in diverse clinical settings. Additionally, the model exhibited lower sensitivity in certain cases, which could limit its ability to identify all high-risk AKI patients. This lower sensitivity may impact its clinical utility in settings where early detection of AKI is critical. Future research should focus on improving sensitivity while maintaining model specificity.

Future studies should also integrate interpretable AI techniques, such as SHAP (SHapley Additive exPlanations), to enhance the transparency of predictions. By providing clear explanations for the model's outputs, these techniques can help clinicians better understand and trust the predictions.

Moreover, prospective validation in various clinical environments is crucial to confirm the practical effectiveness of the XGBoost model. Such validation can help determine the model's robustness across different patient populations and healthcare systems, ensuring its broader applicability and reliability. By conducting validation studies, we can better understand how the model performs in diverse clinical settings and refine it as needed to integrate seamlessly into clinical workflows, ultimately enhancing its utility in everyday clinical practice.

Lastly, the study's reliance on a single type of predictive model (XGBoost) limits the exploration of other potentially effective algorithms. Future studies should consider comparing multiple machine learning approaches to identify the most suitable model for predicting AKI risk.

## Conclusion

In conclusion, this study establishes a deep learning-based prediction model for acute kidney injury (AKI) following off-pump coronary artery bypass grafting (OPCABG) in patients with coronary heart disease. Our findings underscore the importance of accurately identifying high-risk patients and demonstrate the potential of machine learning models in improving AKI prediction. However, further research is essential to optimize and validate these models across diverse clinical settings. Continued efforts in refining these models and integrating them into clinical practice could lead to improved patient outcomes, reduced complications, and ultimately enhance the overall prognosis for patients undergoing OPCABG surgery.

## Data Availability

The original contributions presented in the study are included in the article/Supplementary Material, further inquiries can be directed to the corresponding authors.

## References

[1] Yusuf S Zucker D Peduzzi P Fisher LD Takaro T Kennedy JW Effect of coronary artery bypass graft surgery on survival: overview of 10-year results from randomised trials by the coronary artery bypass graft surgery trialists collaboration. Lancet. (1994) 344(8922):563–70. 10.1016/S0140-6736(94)91963-1 7914958

[2] Chen JJ Chang CH Wu VC Chang SH Hung KC Chu PH Long-term outcomes of acute kidney injury after different types of cardiac surgeries: a population-based study. J Am Heart Assoc. (2021) 10(9):e19718. 10.1161/JAHA.120.019718 PMC8200754 33880935

[3] Wang Y Bellomo R. Cardiac surgery-associated acute kidney injury: risk factors, pathophysiology and treatment. Nat Rev Nephrol. (2017) 13(11):697–711. 10.1038/nrneph.2017.119 28869251

[4] Huang C Qiu J Fang X. Prediction of acute kidney injury after coronary artery bypass graft from preoperative Serum uric acid. J Cardiothorac Vasc Anesth. (2024) 38(10):2247–53. 10.1053/j.jvca.2024.04.018 38890081

[5] Bauer TM Fliegner M Hou H Daramola T Mccullough JS Fu W The relationship between discharge location and cardiac rehabilitation use after cardiac surgery. J Thorac Cardiovasc Surg. (2024) 169(5):1513–21.e6. 10.1016/j.jtcvs.2024.03.024 38522574

[6] Deng Y Ma B Wang X Fan X Nie Q Gao X Association between nadir hematocrit and severe acute kidney injury after off-pump coronary artery bypass graft surgery: a retrospective cohort study based on the MIMIC-IV database. Med Sci Monit. (2022) 28:e937878. 10.12659/MSM.937878 36324246 PMC9641987

[7] Wei Y Zhang Q Wang Z Gong J Chang Q. The effect of preoperative and postoperative glycemic control on acute kidney injury after off-pump coronary artery bypass grafting: a case-control study. Heart Surg Forum. (2022) 25(3):E417–24. 10.1532/hsf.4685 35787755

[8] Zeng J Su X Lin S Li Z Zhao Y Zheng Z. Cardiac surgery-specific subtle perioperative Serum creatinine change in defining acute kidney injury after coronary surgery. JACC Adv. (2024) 3(11):101326. 10.1016/j.jacadv.2024.101326 39493313 PMC11530901

[9] Rao SN Shenoy MP Gopalakrishnan M Kiran BA. Applicability of the Cleveland clinic scoring system for the risk prediction of acute kidney injury after cardiac surgery in a south Asian cohort. Indian Heart J. (2018) 70(4):533–7. 10.1016/j.ihj.2017.11.022 30170649 PMC6116709

[10] Alhulaibi AA Alruwaili AM Alotaibi AS Alshakhs FN Alramadhan HS Koudieh MS. Validation of Various prediction scores for cardiac surgery-associated acute kidney injury. J Saudi Heart Assoc. (2022) 34(4):222–31. 10.37616/2212-5043.1322 36816793 PMC9930984

[11] Jia T Xu K Bai Y Lv M Shan L Li W Machine-learning predictions for acute kidney injuries after coronary artery bypass grafting: a real-life muticenter retrospective cohort study. BMC Med Inform Decis Mak. (2023) 23(1):270. 10.1186/s12911-023-02376-0 37996844 PMC10668365

[12] Flamholz ZN Crane-Droesch A Ungar LH Weissman GE. Word embeddings trained on published case reports are lightweight, effective for clinical tasks, and free of protected health information. J Biomed Inform. (2022) 125:103971. 10.1016/j.jbi.2021.103971 34920127 PMC8766939

[13] Nashef SA Roques F Sharples LD Nilsson J Smith C Goldstone AR EuroSCORE II. Eur J Cardiothorac Surg. (2012) 41(4):734–44. 744–745. 10.1093/ejcts/ezs043 22378855

[14] Hou X Zhang K Liu T Xu S Zheng J Li Y Prediction of acute kidney injury following isolated coronary artery bypass grafting in heart failure patients with preserved ejection fraction using machine leaning with a novel nomogram. Rev Cardiovasc Med. (2024) 25(2):43. 10.31083/j.rcm2502043 39077338 PMC11263137

[15] Shao J Liu F Ji S Song C Ma Y Shen M Development, external validation, and visualization of machine learning models for predicting occurrence of acute kidney injury after cardiac surgery. Rev Cardiovasc Med. (2023) 24(8):229. 10.31083/j.rcm2408229 39076716 PMC11266781

[16] Verma A Sanaiha Y Hadaya J Maltagliati AJ Tran Z Ramezani R Parsimonious machine learning models to predict resource use in cardiac surgery across a statewide collaborative. JTCVS Open. (2022) 11:214–28. 10.1016/j.xjon.2022.04.017 36172420 PMC9510828

[17] Lee HC Yoon HK Nam K Cho YJ Kim TK Kim WH Derivation and validation of machine learning approaches to predict acute kidney injury after cardiac surgery. J Clin Med. (2018) 7(10):322. 10.3390/jcm7100322 30282956 PMC6210196

[18] Thongprayoon C Hansrivijit P Bathini T Vallabhajosyula S Mekraksakit P Kaewput W Predicting acute kidney injury after cardiac surgery by machine learning approaches. J Clin Med. (2020) 9(6):1767. 10.3390/jcm9061767 32517295 PMC7355827

[19] Sun Y Li H. Data mining for evaluating the ecological compensation, static and dynamic benefits of returning farmland to forest. Environ Res. (2021) 201:111524. 10.1016/j.envres.2021.111524 34144010

[20] Jeon D Chavda S Rennert-May E Leal J. Clinical prediction tools for identifying antimicrobial-resistant organism (ARO) carriage on hospital admissions: a systematic review. J Hosp Infect. (2023) 134:11–26. 10.1016/j.jhin.2023.01.003 36657490

[21] Sun H Depraetere K Meesseman L De Roo J Vanbiervliet M De Baerdemaeker J A scalable approach for developing clinical risk prediction applications in different hospitals. J Biomed Inform. (2021) 118:103783. 10.1016/j.jbi.2021.103783 33887456

[22] Quinn KN Wilber H Townsend A Sethna JP. Chebyshev approximation and the global geometry of model predictions. Phys Rev Lett. (2019) 122(15):158302. 10.1103/PhysRevLett.122.158302 31050537

[23] Zhang Y Li L Li Y Zeng Z. Machine learning model-based risk prediction of severe complications after off-pump coronary artery bypass grafting. Adv Clin Exp Med. (2023) 32(2):185–94. 10.17219/acem/152895 36226692

[24] Khanh LN Nguyen QH Chen X Rahardja S Nguyen BP. Classification of adaptor proteins using recurrent neural networks and PSSM profiles. BMC Genomics. (2019) 20(Suppl 9):966. 10.1186/s12864-019-6335-4 31874633 PMC6929330

[25] Susianti H Asmoro AA Sujarwoto Jaya W Sutanto H Kusdijanto AY Acute kidney injury prediction model using cystatin-C, Beta-2 microglobulin, and neutrophil gelatinase-associated lipocalin biomarker in sepsis patients. Int J Nephrol Renovasc Dis. (2024) 17:105–12. 10.2147/IJNRD.S450901 38562530 PMC10984190

[26] Xu L Li C Zhang J Guan C Zhao L Shen X Personalized prediction of mortality in patients with acute ischemic stroke using explainable artificial intelligence. Eur J Med Res. (2024) 29(1):341. 10.1186/s40001-024-01940-2 38902792 PMC11188208

[27] Zeng X Shi S Sun Y Feng Y Tan L Lin R A time-aware attention model for prediction of acute kidney injury after pediatric cardiac surgery. J Am Med Inform Assoc. (2022) 30(1):94–102. 10.1093/jamia/ocac202 36287639 PMC9748588

[28] Wang Y Lu Y Liu C Xiao J. Association between serum calcium level and the risk of acute kidney injury in patients with acute myocardial infarction: evidences from the MIMIC-IV database. Ren Fail. (2024) 46(2):2401137. 10.1080/0886022X.2024.2401137 39252174 PMC11389642

[29] Ahn SW Kim TY Lee S Jeong JY Shim H Han YM Adrenal insufficiency presenting as hypercalcemia and acute kidney injury. Int Med Case Rep J. (2016) 9:223–6. 10.2147/IMCRJ.S109840 27536162 PMC4973717

[30] Hawkins RB Raymond SL Stortz JA Horiguchi H Brakenridge SC Gardner A Chronic critical illness and the persistent inflammation, immunosuppression, and catabolism syndrome. Front Immunol. (2018) 9:1511. 10.3389/fimmu.2018.01511 30013565 PMC6036179

