# Supplementary material for: Deep learning-based prediction model of acute kidney injury following coronary artery bypass grafting in coronary heart disease patients: a multicenter clinical study from China
Source: Front Cardiovasc Med. 2025 Jun 23;12:1600012. doi: 10.3389/fcvm.2025.1600012 (PMC12230002; doi:10.3389/fcvm.2025.1600012)
Supplement: Supplementary file 1 [file Datasheet1.pdf]

Supplementary Table 1. Baseline characteristics of modeling group and validation groups

Variables	Training dataset	Internal validation	P value	External validation dataset	P value
Number	2129	913		878	
Age (years)	62.4 ± 8.78	63.19 ± 8.53	0.612	63.75 ± 8.29	0.73
Male (n, %)	1587 (74.54)	667 (73.06)	0.391	632 (71.98)	0.275
Hypertension (n, %)	1421 (66.74)	618 (67.69)	0.614	555 (63.22)	0.143
Diabetes (n, %)	825 (38.75)	376 (41.18)	0.21	398 (45.33)	0.007
CVD (n, %)	249 (11.70)	124 (13.58)	0.148	132 (15.03)	P<0.001
CBP (n, %)	377 (17.71)	167 (18.29)	0.756	128 (14.58)	0.074
Angina (n, %)	1942 (91.22)	838 (91.79)	0.725	808 (92.03)	0.799
COPD (n, %)	47 (2.21)	21 (2.31)	0.894	19 (2.16)	0.562
BMI (kg/m^2)	25.78 ± 3.2	25.68 ± 3.16	0.252	26.08 ± 3.28	0.221
Preoperative Scr	74.68 ± 29.03	75.18 ± 28.97	0.421	71.24 ± 30.14	0.245
Preoperative LVEF	58.43 ± 9.17	58.06(9.53)	0.158	58.63 ± 8.69	0.108
TG	1.63 ± 1.06	1.68 ± 0.93	0.37	1.66 ± 1.27	0.143
TC	3.93 ± 1.01	3.98 ± 1.05	0.8	3.97 ± 1.03	0.617
Hemoglobin	116.96 ± 23.38	117.39 ± 23.53	0.522	111.93 ± 24.2	0.325
HDL-C	0.97 ± 0.23	1.0 ± 0.24	0.248	1.06 ± 0.56	0.001
LDL-C	2.31 ± 0.84	2.33 ± 0.89	0.265	2.43 ± 0.98	0.009
Urea	6.82 ± 3.32	6.96 ± 3.31	0.296	6.55 ± 2.76	0.105
UA	335.13 ± 89.94	333.07 ± 96.67	0.219	332.74 ± 97.75	0.009
Alb	41.87 ± 3.53	41.73 ± 3.58	0.579	41.68 ± 4.91	0.001
TP	68.43 ± 5.62	68.48 ± 5.64	0.593	67.45 ± 6.23	0.001
T-Bil	12.33 ± 5.9	12.03 ± 5.45	0.223	12.61 ± 6.61	0.201
D-Bil	3.92 ± 2.33	3.86 ± 2.04	0.175	4.23 ± 3.02	0.025
WBC	9.95 ± 4.08	9.75 ± 3.92	0.148	9.92 ± 4.62	0.876
RBC	3.81 ± 0.75	3.84 ± 0.77	0.403	3.68 ± 0.81	0.029
PLT	211.15 ± 75.03	214.16 ± 78.44	0.269	210.42 ± 76.4	0.448
Neu	74.26 ± 12.64	73.79 ± 12.73	0.296	74.81 ± 13.2	0.198
Glu	8.15 ± 3.46	8.17 ± 3.42	0.828	7.95 ± 3.47	0.235
Ca	2.23 ± 0.16	2.23(0.16)	0.188	2.27 ± 1.03	0.071
ALP	82.24 ± 36.78	81.94 ± 30.26	0.703	81.16 ± 68.23	0.053
Hb	114.11±42.17	117.24±39.45	0.614	112.25±46.34	0.091
CK-MB	7.56±3.27	6.97±4.19	0.571	7.14±3.99	0.124
HCT	34.07±5.31	36.21±6.11	0.447	39.11±8.71	0.081

Abbreviation: CVD: Cardiovascular Disease, CBP: Chronic Bronchitis, COPD: Chronic Obstructive Pulmonary Disease, BMI: Body Mass Index, Preoperative Scr: Preoperative Serum Creatinine, Preoperative LVEF: Preoperative Left Ventricular Ejection Fraction, TG: Triglycerides, TC: Total Cholesterol, HDL-C: High-Density Lipoprotein Cholesterol, LDL-C: Low-Density Lipoprotein Cholesterol, UA: Uric Acid, Alb: Albumin, TP: Total Protein, T-Bil: Total Bilirubin, D-Bil: Direct Bilirubin, WBC: White Blood Cell Count, RBC: Red Blood Cell Count, PLT: Platelet Count, Neu: Neutrophil Count, Glu: Glucose, Ca: Calcium, ALP: Alkaline Phosphatase, AKI: Acute Kidney Injury, Hb: Hemoglobin ,CK-MB: Creatine Kinase-MB, HCT: Hematocrit.

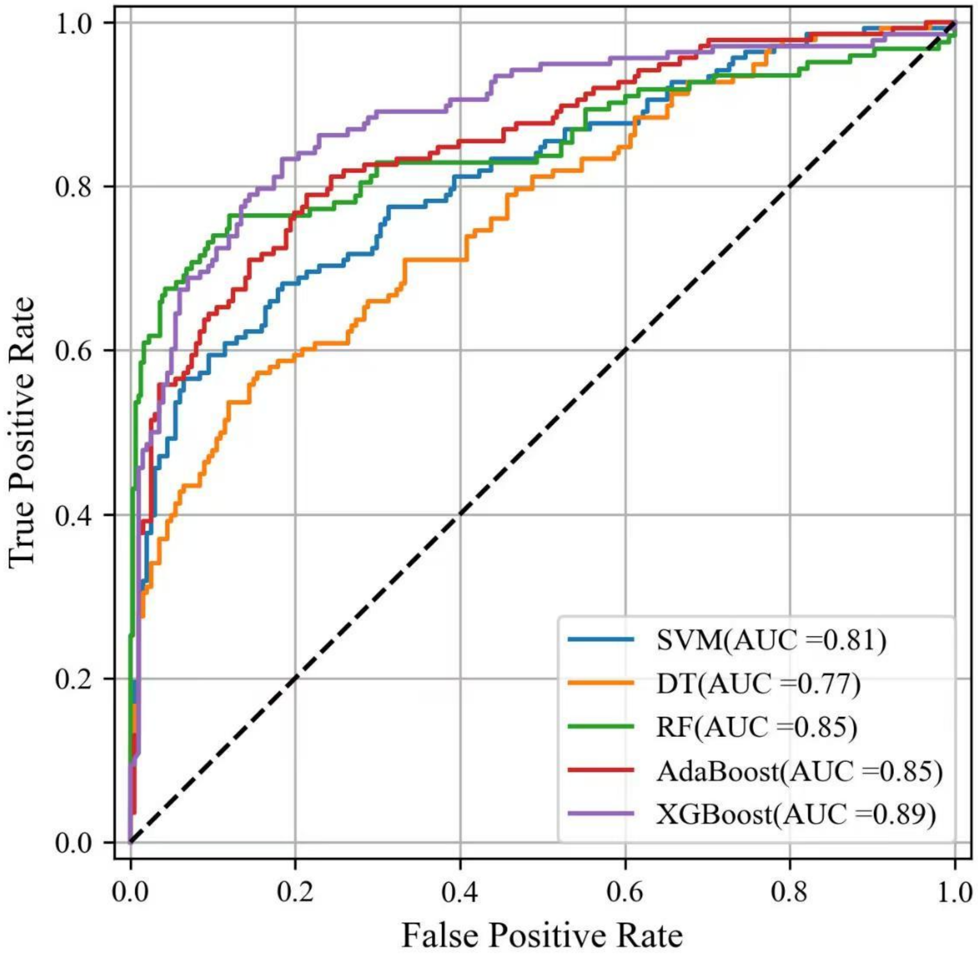

Supplementary Figure 1. Validation of Internal Dataset Using Five Machine Learning Models

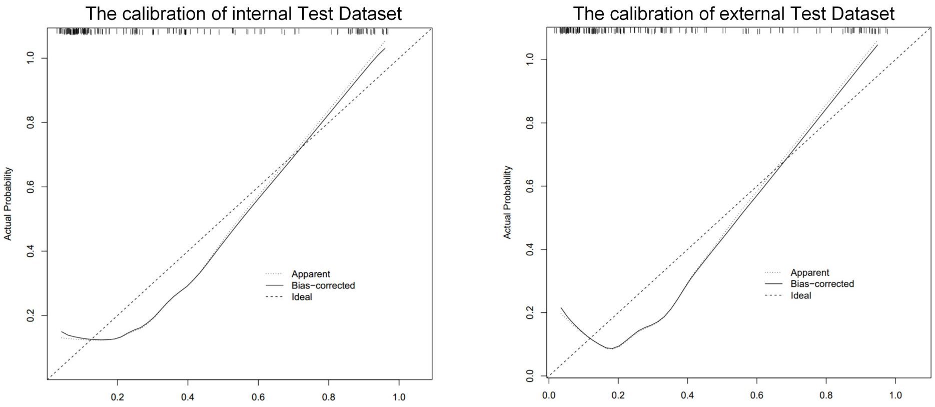

Supplementary Figure 2. The calibration of XGBoost model.
